# Exploration of the Role of Relationships and Virtual Learning on Academic Performance and Mental Health

**DOI:** 10.7759/cureus.28338

**Published:** 2022-08-24

**Authors:** Deepal Patel, Shaun Andersen, Genesis Leon, Cynthia Lee, Edward Simanton

**Affiliations:** 1 Medical Education, University of Nevada Las Vegas School of Medicine, Las Vegas, USA; 2 Office of Medical Education, University of Nevada Las Vegas, Las Vegas, USA

**Keywords:** covid-19, medical school curriculum, medical students, online learning, student relationships, mental health, academic performance, medical education

## Abstract

Background

The coronavirus disease 2019 (COVID-19) pandemic caused medical schools to rapidly transition to online/distance learning. Online learning is often associated with poor academic performance, mental health, and student-to-faculty relationships. The purpose of this study is to determine if correlations exist between academic performance, mental health, study location, and student/faculty relationships among medical students.

Methodology

First-year medical students received a survey asking them to reflect on their study location, mental health, and student/faculty relationships during the COVID-19 pandemic. Second- and third-year medical students received a similar survey asking them to reflect on their experiences from the perspective of their first year of medical school (pre-pandemic). The first five exam scores were gathered for all participants. Pearson’s correlation coefficient was calculated between all variables.

Results

Academic performance was found to be positively correlated with both mental health (R = 0.215, p = 0.016) and relationships among students (R = 0.0259, p = 0.004), while negatively correlated with the percentage of time spent studying at home (R = -0.185, p = 0.039). Mental health was additionally found to be positively correlated with relationships to faculty (R = 0.230, p = 0.01) and relationships to students (R = 0.245, p = 0.006).

Conclusions

Academic performance and mental health are correlated with relationships and study location. These correlations may explain the negative outcomes associated with online learning in medical education.

## Introduction

The coronavirus disease 2019 (COVID-19) pandemic has caused many medical schools to rapidly pivot from traditional, in-person education to online learning [1,2]. Studies show that this change has led to students spending more time studying at home and less time studying at the school campus. Although online learning has been used in many aspects of medical education prior to COVID-19, isolation due to distance learning as a consequence of the pandemic has contributed to students’ stress and has also increased mental health concerns [3,4]. Additionally, online learning is shown to have a negative impact on student-to-student and student-to-faculty communication and relationships [4-7].

Medical schools use exams to assess students’ understanding of the material. Studies show that university students who have had the opportunity to attend live lectures perform better on exams than those in the online classroom (distance learning) [8,9]. Furthermore, a study by Wayne et al. evaluated medical students’ academic performance with the school’s learning environment and found that students who felt that the program had high student-to-student interaction performed better on exams [10]. Studies have associated increased faculty teaching and tutoring (e.g., office hours) with increased student academic performance [11,12]. However, there is a lack of literature that assesses the correlation between students’ general perception of their relationship with their faculty and academic performance. Student mental health has also been linked to academic performance. Students who had mental health concerns performed and functioned poorer academically [13-16].

Some studies suggest that medical students may have poorer mental health ratings compared to the general population [17-20]. Krane et al. found that, in general, student-to-faculty relationships were positively correlated with better mental health ratings [21]. Furthermore, Yin et al. showed that students with low student-to-student and student-to-faculty support were three times more likely to experience depressive or anxiety-related symptoms compared to students with high social support ratings [22]. Although both mental health and in-person attendance have been linked to academic performance, the relationship between mental health and study location has not been well documented.

Because of the COVID-19 pandemic, live lectures across all levels of education were transitioned to a virtual format, leading students to study less at school and more on their own at home. This change presented a natural barrier to student-to-student and student-to-faculty interactions. This study aims to determine if correlations exist between academic performance, mental health, student/faculty relationships, and study location among medical students.

## Materials and methods

Study subjects and setting

In 2020, the first-year class from the Kirk Kerkorian School of Medicine (KKSOM) at the University of Nevada Las Vegas (UNLV) transitioned to virtual education due to the COVID-19 pandemic. Prior classes, however, naturally experienced fundamental aspects of their first-year medical school transition such as live lectures and group studying on campus and in local libraries. Spending large amounts of time together in person allowed the students to develop camaraderie with their peers and school faculty. In contrast, the 2020 first-year class had a distinctly different experience in which they had much less interaction with their peers and faculty members due to the cancellation of select classes and the abrupt transition to online learning.

Deidentified data for this study were drawn from an institutional repository in compliance with an approved Institutional Review Board protocol, number 1030906-1, “School of Medicine use of program evaluation data for research.” We obtained access to an exam performance database using this protocol. The second source of deidentified data for this study was the survey sent out to students. Further, this survey aimed to supply school leaders and teaching faculty with insight to evaluate curriculum adjustments made in response to the COVID-19 pandemic and their impacts on the student experience.

Survey

First-year medical students were presented with a Qualtrics survey to obtain information about their experiences during the first semester of medical school. Second- and third-year students were also presented with a similar survey about their experiences during their first semester of medical school which was not affected by the pandemic. The primary difference between surveys was that first-year medical students were specifically asked about how COVID-19 impacted their education, while second- and third-year students were asked to reflect on their first semester prior to the pandemic. Both surveys primarily asked students about their study locations (campus vs. home) and relationships with their peers and faculty members. The surveys also asked students about their mental health during this time. Mental health, relationships with faculty, and relationships with students were all reported using a 1-5 Likert scale. Time spent studying in each location (at home vs. at campus) was reported in percentages of total time spent studying. Surveys also included an optional free response text box for participants to further elaborate on their answers. Particularly relevant responses are indicated in the Results section where appropriate. The survey response rate was as follows: 47 out of 58 first-year students, 45 out of 60 second-year students, and 33 out of 60 third-year students. This process amounted to a convenience sample where all participants were included for data analysis. Both quantitative and qualitative responses (free response) were gathered and are appropriately summarized in the Results section.

Data analysis

To assess students’ academic performance, the first five exams taken during the first semester of medical school were used. These exams are created using faculty-selected standardized questions from the National Board of Medical Examiners (NBME). Each exam was administered to align with topics pertaining to the basic medical sciences (e.g., biochemistry, virology), as well as organ systems (e.g., neurology, musculoskeletal). Scores for each exam were compared against the national mean: above the mean provides a 1.0 (100%) and below the mean provides a 0.0 (0%). As such, the academic performance metric is calculated as a mean of those five values for each student. Averaging these values yields the NBME-alpha reported in the Results section.

Survey responses were analyzed in the following manner: each study location response, home or campus, was averaged to ascertain a representative time spent per destination. The Likert scores collected from the mental health, relationship to faculty, and relationship to students survey responses were all averaged as well.

## Results

Descriptive statistics of study variables (mean, standard deviation, minimum, maximum) are shown in Table 1.

**Table 1:** Descriptives of the surveyed population (mean, standard deviation, minimum, maximum). NBME: National Board of Medical Examiners

Study variables	Mean	Standard deviation	Minimum	Maximum
NBME-alpha	68.88%	27.57%	0.20	1.00
Percentage of time spent studying at home	68.35%	31.44%	0.00	100.00
Percentage of time spent studying at campus	21.62%	25.70%	0.00	100.00
Mental health (1–5)	3.30	1.18	1.00	5.00
Relationship to faculty (1–5)	2.73	1.27	1.00	5.00
Relationship to students (1–5)	3.42	1.24	1.00	5.00

Note that both location variables (studying at home and studying at campus) do not add up to 100%. This discrepancy is due to a third study option presented in the survey (“elsewhere”), representing other locations students may have studied at, such as libraries, coffee shops, etc. Relationships with faculty in comparison to the other variables measured on the Likert scale were especially low. A representative testimonial is shown below:

“COVID has also severely limited my opportunities to connect and network with other students and faculty….”

This study initially examined academic performance and variables associated with performance. Pearson correlation coefficients between the academic performance variable (NBME-alpha) and other study variables are shown in Table 2.

**Table 2:** Academic performance correlations (Pearson correlation coefficient (r), p-value). *Statistically significant with an alpha of 0.05. NBME: National Board of Medical Examiners

Variables correlated with academic performance	Pearson correlation coefficient (r)	P-value
NBME-alpha	0.215	0.016*
Percentage of time spent studying at home	-0.034	0.703
Percentage of time spent studying at campus	-0.012	0.896
Relationship to faculty (1–5)	0.230	0.01*
Relationship to students (1–5)	0.245	0.006*

As shown in Table 2, a significant negative correlation exists between academic performance and the percentage of time spent studying at home (R = -0.185, p = 0.039), suggesting poorer performance with greater time spent home studying.

Relationship with students was the variable most strongly correlated with academic performance (R = 0.259, p = 0.004). Other significant positive correlations with academic performance are mental health (R = 0.215, p = 0.016) and studying on campus (R = 0.175, p = 0.050), indicating the beneficial impact these measures also have for student academic performance. These significant findings are shown below in figures to illustrate the implication of the correlations.

**Figure 1:**
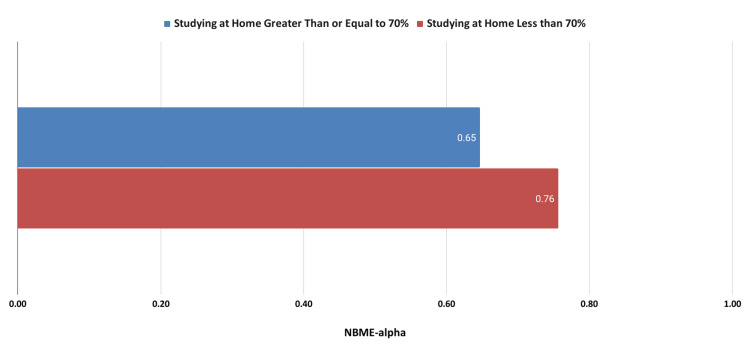
Academic performance comparing study locations. NBME: National Board of Medical Examiners

The academic performance of students who studied less than 70% of the time at home compared with the performance of students who studied at home at least 70% of the time is depicted in Figure 1.

As shown in Figure 1, students who studied at home at least 70% of the time performed poorer on their NBME examinations. Representative testimonials are shown below:

“COVID has taken away so many components from our educational experience and enrichment....”

“...I’m someone who learns best from discussing in a small group and I live alone, so being at home was very isolating.”

**Figure 2:**
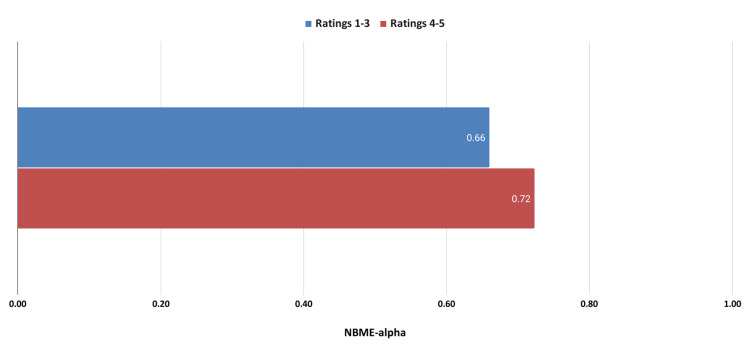
Academic performance comparing higher and lower mental health ratings. NBME: National Board of Medical Examiners

Academic performance comparisons were made between students who rated their mental health 1-3 on the survey versus students with mental health ratings of 4 and 5. These comparisons are shown in Figure 2.

Figure 2 shows students with higher mental health ratings achieved better academic performance. A representative testimonial is shown below:

“It has made other things I like doing outside of school either limited or non-existent, which has made school seem that much more overbearing.”

**Figure 3:**
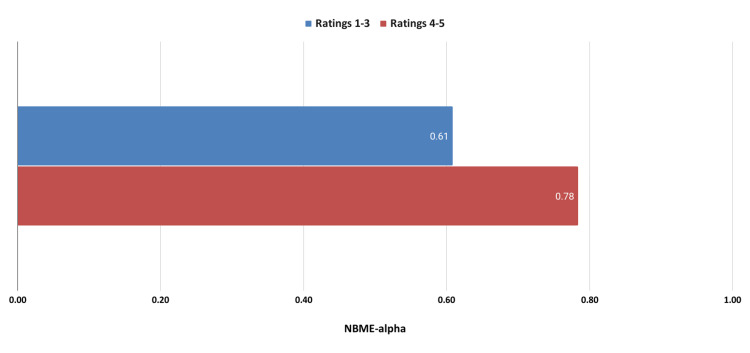
Academic performance comparing higher and lower student relationship ratings. NBME: National Board of Medical Examiners

Table 2 shows that relationships between students had the strongest correlation to academic performance. In Figure 3, we compared students who rated relationships with students as 1-3 on the survey with students who rated these relationships as 4 or 5.

As shown in Figure 3, students with higher relationship ratings achieved higher academic performance. A representative testimonial is shown below:

“My first year of medical school has been an all-around awful experience, 100% due to the virtual reality of COVID. I barely know most of my classmates.”

Second, we assessed the links to mental health. Pearson correlation coefficients between the mental health variable and other study variables are shown in Table 3.

**Table 3:** Mental health correlations (Pearson correlation coefficient (r), p-value). *Statistically significant with an alpha of 0.05. NBME: National Board of Medical Examiners

Variables correlated with mental health	Pearson correlation coefficient (r)	P-value
NBME-alpha	0.215	0.016*
Percentage of time spent studying at home	-0.034	0.703
Percentage of time spent studying at campus	-0.012	0.896
Relationship to faculty (1–5)	0.230	0.01*
Relationship to students (1–5)	0.245	0.006*

Mental health is significantly associated with academic performance (as noted above in Table 3), as well as with relationships to faculty (R = 0.230, p = 0.01) and students (R = 0.245, p = 0.006), suggesting these three variables have helpful effects on student mental health. A representative testimonial is shown below:

“When it comes to knowing faculty, I've watched the classes above us interact with faculty and I do not feel as though I even have half of a relationship with anyone.”

Although most students found the isolation of COVID-19 to be a negative impact on mental health, some students preferred the freedom from the structure that virtual education provided. Representative testimonials are shown below:

“COVID has allowed me to focus more on my studies, my hobbies, and my family. I have been very happy.”

“I was thankful to be able to be remote. It allowed me to help out my family (who don't live in Vegas) with grocery shopping and other chores in order to reduce their risk of getting COVID….” 

Additional findings are reported in Table 4; however, these findings are outside the central scope of this study.

**Table 4:** All variables with correlations (NBME-alpha, percentage of time spent studying at home, percentage of time spent studying at campus, mental health (1-5), relationship to faculty (1-5), relationship to students (1-5)). *Statistically significant with an alpha of 0.05. NBME: National Board of Medical Examiners

	NBME-alpha		Percentage of time spent studying at home		Percentage of time spent studying at campus		Mental health (1–5)		Relationship to faculty (1–5)		Relationship to students (1–5)	
	Pearson correlation	P-value	Pearson correlation	P-value	Pearson correlation	P-value	Pearson correlation	P-value	Pearson correlation	P-value	Pearson correlation	P-value
NBME-alpha	1	---	-0.185	0.039*	0.175	0.050	0.215	0.016*	0.166	0.064	0.259	0.004*
Percentage of time spent studying at home	-0.185	0.039*	1	---	-0.804	<0.001*	-0.034	0.703	-0.346	<0.001*	-0.525	<0.001*
Percentage of time spent studying at campus	0.175	0.050	-0.804	<0.001*	1	---	-0.012	0.896	0.305	0.001*	0.466	<0.001*
Mental health (1–5)	0.215	0.016*	-0.034	0.703	-0.012	0.896	1	---	0.230	0.01*	0.245	0.006*
Relationship to faculty (1–5)	0.166	0.064	-0.346	<0.001*	0.305	0.001*	0.230	0.01*	1	---	0.655	<0.001*
Relationship to students (1–5)	0.259	0.004*	-0.525	<0.001*	0.466	<0.001*	0.245	0.006*	0.655	<0.001*	1	---

Having a higher-rated student relationship with faculty is positively correlated with time spent studying at the campus (R = 0.305, p = 0.001), mental health (R = 0.230, p = 0.01), and relationship with students (R = 0.655, p < 0.001). However, a negative association exists between the relationship with faculty and the percentage of time spent studying at home (R = -0.346, p < 0.001). While there may be a baseline level of digital interaction between students and faculty through virtual education, increasing time spent studying at home does not necessitate improved relationships among both groups.

Relationships with students had the greatest number of significant associations with each of the variables collected. Positive correlations were also seen with the percentage of time spent studying at the campus (R = -0.466, p < 0.001). A negative correlation was also found with the percentage of time spent studying at home (R = -0.525, p < 0.001).

## Discussion

The findings of this study showed significant relationships between academic performance and time spent studying at home, mental health, and student-to-student relationships. Academic performance was not found to be related to student-to-faculty relationships. Mental health was also shown to be correlated with student-to-student and student-to-faculty relationships but not with study location.

Academic performance

The strongest correlation in this study was between student-to-student relationships and academic performance. Studies have found these student relationships to be crucial in Step 1 preparation [10]. However, our findings suggest that this is also important earlier in medical school. We suggest that programs should emphasize student bonding, networking, and team building during the first year of medical school to maximize academic performance. Our work also suggests that studying at home can be detrimental to academic performance. This should be expected because home study limits the likelihood of developing student-to-student relationships. Studying in group settings rather than at home allows all students to come to a consensus regarding the wise use of study material: whether that may be what is agreed to be high-yield to maximize group time or what has been prioritized for discussion. At this time, there is a paucity of literature that assesses the optimization of home-learning environments. We suggest that schools encourage students to study outside of the home. If stay-at-home orders are necessary, such as in the case of a global pandemic, then programs should explore alternative ways to facilitate student relationship-building online. Although relationships between students are related to academic success, students’ perceived relationships with their faculty were not significantly correlated with academic performance. However, previous research suggests that teachers should provide enough learning experiences through lectures, tutoring, and office hours to ensure student success [12,13].

Students who rated their mental health higher tended to have better academic performance. We find this to be consistent with the study conducted by Bruffaerts et al. in which worse mental health scores were found to be linked with impaired academic function [13]. Though our study used self-reported mental health ratings, other studies have assessed the relationships between academic performance and specific mental health conditions such as depression [15]. This phenomenon is also evidenced in the collected student testimonials. To this end, educators should check in with their students and make sure they have adequate access to mental health support. This may be especially important for students who are performing poorly. Such efforts would be prudent and act as a protective measure for students who may be struggling with unreported mental health concerns and are at risk of academic probation or suspension.

Mental health

Student ratings of mental health were found to be correlated with both student-to-faculty and student-to-student relationships. This finding agrees with a review by Krane et al. indicating that faculty and peer support had significant positive effects, broadly speaking, on depression and student self-esteem [21]. Such support may combat findings from other studies that suggest medical students suffer from poorer mental health than the general student population [17-20]. Given that the transition to medical school is a difficult time for many students, faculty ought to foster and develop relationships among their students and themselves. Likewise, medical students should make efforts to form study groups and friendships with their peers.

Our results show that mental health ratings were not correlated with study location. Within the context of our study, it seems mental health is most strongly influenced by the interpersonal networks built within the medical school. As such, building those aforementioned relationships is a priority, irrespective of study location. Educators should utilize small-group, interactive activities in both in-person and online settings to ensure that students are allowed proper interaction time with both peers and faculty members. Furthermore, instructors should regularly follow up with students to build and facilitate good rapport so students feel comfortable reaching out for any mental health or learning concerns.

This study has determined that student relationships are strongly correlated with both academic success and mental health, but study location only impacts academic performance. A deeper examination into the nuances of relationships as they impact academic performance and mental health in medical schools and other educational settings is warranted. While this study is consistent with the results of prior work, further efforts should be made to examine other factors that make up the medical student experience.

Limitations

Our study has limitations worth mentioning. First, the data were collected from a single medical institution, limiting the generalizability of these findings to other medical schools, as well as other levels of education. Additionally, the survey responses are contingent on students’ ability to recall their experiences and feelings accurately. Furthermore, the mental health ratings are subject to a self-reporting bias. This study yielded many other compelling associations that are outside the scope of this paper (Table 4). However, we limited our scope to only those variables that lacked obvious confounds to avoid intentional data dredging. This was a matter of author consensus: for example, we chose not to report the correlation between studying at home and studying on campus due to the obvious negative relationship between these two variables. Future work may be taken to investigate further analysis and reference to the literature regarding other unreported yet compelling correlations.

## Conclusions

There may be benefits to the social network developed among students and faculty within the context of the traditional in-person model of medical education, especially when it pertains to academic performance and mental health. These two variables, in particular, have a synergistic relationship that should be prioritized when designing medical school curricula. Although our suggestions may be difficult to implement in the setting of virtual education, they are paramount to medical student success and well-being.
